# Path-enhanced graph convolutional networks for node classification without features

**DOI:** 10.1371/journal.pone.0287001

**Published:** 2023-06-09

**Authors:** Qingju Jiao, Peige Zhao, Hanjin Zhang, Yahong Han, Guoying Liu

**Affiliations:** 1 School of Computer and Information Engineering, Anyang Normal University, and Key Laboratory of Oracle Bone Inscriptions Information Processing, Ministry of Education of China, Anyang, Henan, China; 2 School of Computer and Artificial Intelligence, Zhengzhou University, Zhengzhou, Henan, China; 3 School of Software and Internet of Things Engineering, Jiangxi University of Finance and Economics, Nanchang, Jiangxi, China; 4 College of Intelligence and Computing, Tianjin University, Tianjin, China; 5 School of Software Engineering, Anyang Normal University, and Key Laboratory of Oracle Bone Inscriptions Information Processing, Ministry of Education of China, Anyang, Henan, China; The University of Lahore, PAKISTAN

## Abstract

Most current graph neural networks (GNNs) are designed from the view of methodology and rarely consider the inherent characters of graph. Although the inherent characters may impact the performance of GNNs, very few methods are proposed to resolve the issue. In this work, we mainly focus on improving the performance of graph convolutional networks (GCNs) on the graphs without node features. In order to resolve the issue, we propose a method called *t-hop*GCN to describe *t-hop* neighbors by the shortest path between two nodes, then the adjacency matrix of *t-hop* neighbors as features to perform node classification. Experimental results show that *t-hop*GCN can significantly improve the performance of node classification in the graphs without node features. More importantly, adding the adjacency matrix of *t-hop* neighbors can improve the performance of existing popular GNNs on node classification.

## 1. Introduction

Deep learning models have been successfully applied to different fields, such as computer vision [1], natural language processing [2] and false data injection attack [3]. However, these models do not handle the graph data which can easily describe many real complex systems in biology, physics, sociology and computer science. Graph Neural Networks (GNNs) that combine the paradigm of deep learning can deal with the tasks on graph data [4]. Graph convolutional networks (GCNs) [5] are typical and successful models of GNNs and undergo rapid development over the past few years.

A lot of deuterogenic GCNs are proposed to improve the performance and apply to different fields. Pei et al propose a geometric aggregation scheme (termed Geom-GCN) [6] to improve the performance of GCN. Geom-GCN can overcome the losses of discriminative structures and long-range dependencies by aggregating the structural neighborhoods in latent space. As with Geom-GCN, the method WGCN proposed by Zhao et al embeds graphs into a latent space, and gains geometrical relationships of nodes [7]. To improve the classification accuracy of GCN, Wang et al propose an adaptive multi-channel graph convolutional network (AM-GCN) [8]. AM-GCN employs attention mechanism to learn adaptive weights of the embeddings from nodes features and topological structures. Yang et al propose a factorizable graph convolutional network (FactorGCN) [9] to produce disentangled node features which is used for graph and node classification. FactorGCN disentangles inputted graph into several factorized graphs which correspond to several latents, and aggregates nodes in each latent to produce new features. Using a feature similarity preserving aggregation which can fuse graph structure and node features, SimP-GCN [10] is proposed to improve the performance of GCN. Furthermore, SimP-GCN also can acquire the feature similarity and dissimilarity relations between nodes by self-supervised learning.

The GCN proposed by Kipf et al [5] only uses two convolutional layers, but shallow layers may not capture deeper topology structure and the information of high-order neighbors [11]. But deep GCNs suffer from over smoothing and over fitting. By removing certain edges which makes the connections between nodes more sparse and generates more diversity into the graph, DropEdge [12] proposed by Rong et al can alleviate both over smoothing and over fitting issues in deep GCN. Chen et al employ initial residual and identity mapping to design a deep GCNII model [11]. The model relieves the over smoothing problem on semi and fully supervised tasks. Feng et al propose a graph random neural network (GRAND) [13] to alleviate the over smoothing issue. GRAND first augments graph by a random propagation strategy and then optimizes prediction consistency by consistency regularization. Chen et al propose a residual network structure to resolve over smoothing problem for user-item interaction data [14]. Bo et al propose a frequency adaptation graph convolutional network (FAGCN) which adaptively fuses low-frequency and high-frequency signals to alleviate the over smoothing problem [15]. Yang et al propose multilayer graph convolutional networks with dropout (DGCs) to perform feature augmentation and relieve over fitting problem by performing nonlinearity removal and weight matrix merging between graph conventional layers [16].

Existing graph neural networks (GNNs) may suffer from high time complexity and high demand of memory. Wang et al propose a binary graph convolutional network (Bi-GCN) to handle the issue by binarizing network parameters and node features [17]. By considering the random features in speeding up the training, Huang et al propose a graph convolutional network with random weights (GCN-RW) [18], which employs random filters to revising convolutional layer and regularized least squares loss to adjust learning objective. Graph sampling is a classic and effective model to resolve time and memory challenges. Therefore, some sampling-based GCNs are proposed, such as Cluster-GCN [19] and fastGCN [20]. Although graph topology sampling is an effective method to reduce the memory and computational cost in training GCNs, the study of the relationship between them is rare from the view of theory. Therefore, Li et al describe the impact of generalization performance and sample complexity from graph structures and topology sampling [21].

The GCNs mentioned above are designed from the view of methodology. In fact, the intrinsic characters of graph data (such as incompleteness, noise and dynamic) impact the performance of GCNs. In order to overcome the incompleteness and missing, Taguchi et al use gaussian mixture model to represent missing data and calculate the expected activation of neurons, which enable GCN to resolve the issue mentioned above [22]. Gan et al propose a dynamic graph convolutional network to obtain high-quality data from original graph by fusing multiple local and global graphs, and then perform GCN in a low-dimensional space [23]. Pareja et al propose evolving graph convolutional networks for dynamic graphs (EvolveGCN) [24]. EvolveGCN uses recurrent neural network (RNN) to describe dynamic graphs by evolving network parameters.

Most node classification methods using GNNs work well by aggregating adjacent node features iteratively [25, 26]. However, a large number of graphs do not contain node features. For example, a classical graph without node features is a molecular graph in which nodes and edges represent atoms and chemical bonds respectively [27, 28]. In the social field, the graphs in the REDDIT data [29, 30] also do not include node features. The nodes in these graphs are users and the edges represent the mutual relationship of comments. Unfortunately, current GNNs cannot obtain excellent performance on the graphs without node features [31]. In this study, we focus on the node classification using GCNs without node features. To improve the performance of GCNs without node features, it is necessary to extract more information of adjacent nodes through the graph topology, such as 2*-hop* neighbors or farther hop neighbors. In fact, the message between adjacent nodes is passed along edge paths [12]. To resolve the issue mentioned above, we introduce *t-hop* neighbors [32], which are generated by edge paths as feature matrix of GCNs, to capture more adjacent information. Different from GCNs, the input feature matrix of the proposed method (named *t-hop*GCN) is a *t-hop* adjacency matrix instead of an identity matrix. Experimental results show that the proposed method *t-hop*GCN can significantly improve the performance of node classification in the graph without node features. The main contributions of this study can be summarized as follows. First, we extract a new feature matrix from graph structure by *t-hop* neighbors introduced in this work. The new feature matrix can provide a universal guideline to extract node features from graph information including neighborhoods and path. Second, a novel approach *t-hop*GCN for node classification is proposed, and *t-hop*GCN outperforms other GNNs by a large margin. And finally, the performance of 12 GNNs or variants are improved obviously on the graphs without node features by adding the *t-hop* feature matrix, indicating that our research can be used as a general skill to improve the performance.

The rest of this work is organized as follows. In section 2, we introduce the principles of GCN and the proposed *t-hop*GCN in detail. Section 3 provides the comparative experimental results on six graphs data. Then, the performance of different GNNs on node classification by adding *t-hop* matrix features is investigated in section 4. Section 5 discusses the selection of a parameter in *t-hop*GCN. Finally, Section 6 concludes this work and provides some directions for future works.

## 2. Methods

### 2.1. Graph convolutional networks

Here, we first introduce some basic concepts of a graph. A graph *G* with *n* nodes and *m* edges is described as *G* = (*V*,*E*), *V* and *E* are the sets of nodes and edges in the graph, respectively. If each node *v*_*i*_ has *d* features, the graph can also be represented as *G* = (*V*,*E*,**X**), where $X={\left[x_{1},x_{2},\cdots ,x_{i},\cdots ,x_{n}\right]}^{T}\in R^{n\times d}$ is the feature matrix for *n* nodes, and *x*_*i*_∈*R*^*d*^ is the *d* dimensional feature vector of node *v*_*i*_. To calculate conveniently, a graph is usually written in the form of adjacency matrix **A**. If there is an edge between node *v*_*i*_ and node *v*_*j*_, then **A**(*i*,*j*) is the weight of the edge; otherwise **A**(*i*,*j*) = 0.

GCN is a classic model of GNNs and describes node features by aggregating the features from its neighbors. A main content of GCNs is a layer-wise propagation rule for neural network models described as Eq (1) [5]:

$$H^{\left(l+1\right)}=\sigma ({\tilde{D}}^{-1/2}\tilde{A}{\tilde{D}}^{-1/2}H^{\left(l\right)}W^{\left(l\right)})$$ (1)

**H**^(*l*)^ is the matrix of activations in the *l*^*th*^ layer; **H**^(0)^ = **X**, σ(⋯) denotes an activation function, such as the *ReLU*(⋯) = *max* (0,⋯); $\tilde{A}=A+I$ (**I** is an identity matrix) and $\tilde{D}$ is a degree matrix, ${\tilde{D}}_{ii}=\sum_{j=1}^{n}{\tilde{A}}_{ij};\;W^{\left(l\right)}\in R^{d\times f}$ with *d* dimensional feature vector and *f* filters is a trainable weight matrix in layer *l*.

The graph convolutional network is applied for semi-supervised classification by a two-layer graph convolutional networks with a softmax (Eq (2)) classifier on the output features.

$$Z=softmax(\hat{A}ReLU(\hat{A}XW^{\left(0\right)})W^{\left(1\right)})$$ (2)

where $\hat{A}={\tilde{D}}^{-1/2}\tilde{A}{\tilde{D}}^{-1/2},\;softmax(x_{i})=\frac{1}{\sum_{i}exp(x_{i})}exp(x_{i})$, the weights **W**^(0)^ and **W**^(1)^ are trained using gradient descent.

The loss function is defined as the cross-entropy loss over all labeled nodes (Eq (3)):

$$L=-\sum_{l\in y_{L}}\sum_{f=1}^{F}Y_{lf}\;\ln\;Z_{lf}$$ (3)

where *y*_*L*_ is the set of node indices with labels, *F* is the dimension of the output features and is equal to the number of classes. $Y\in R^{|y_{L}|\times F}$ is a label indicator matrix.

### 2.2. Our method *t-hop*GCN

We first introduce the *t-hop* (or *t-order*) neighbors ($N_{i}^{t}$) of a node *v*_*i*_ by edges path like the node’s local neighborhood defined by Hamilton [33]. For a node *v*_*i*_, $N_{i}^{t}$ is the set of nodes whose shortest path (*d*_*sp*_) to node *v*_*i*_ is less than or equal to *t* (see Eq (4)).

**Fig 1:**
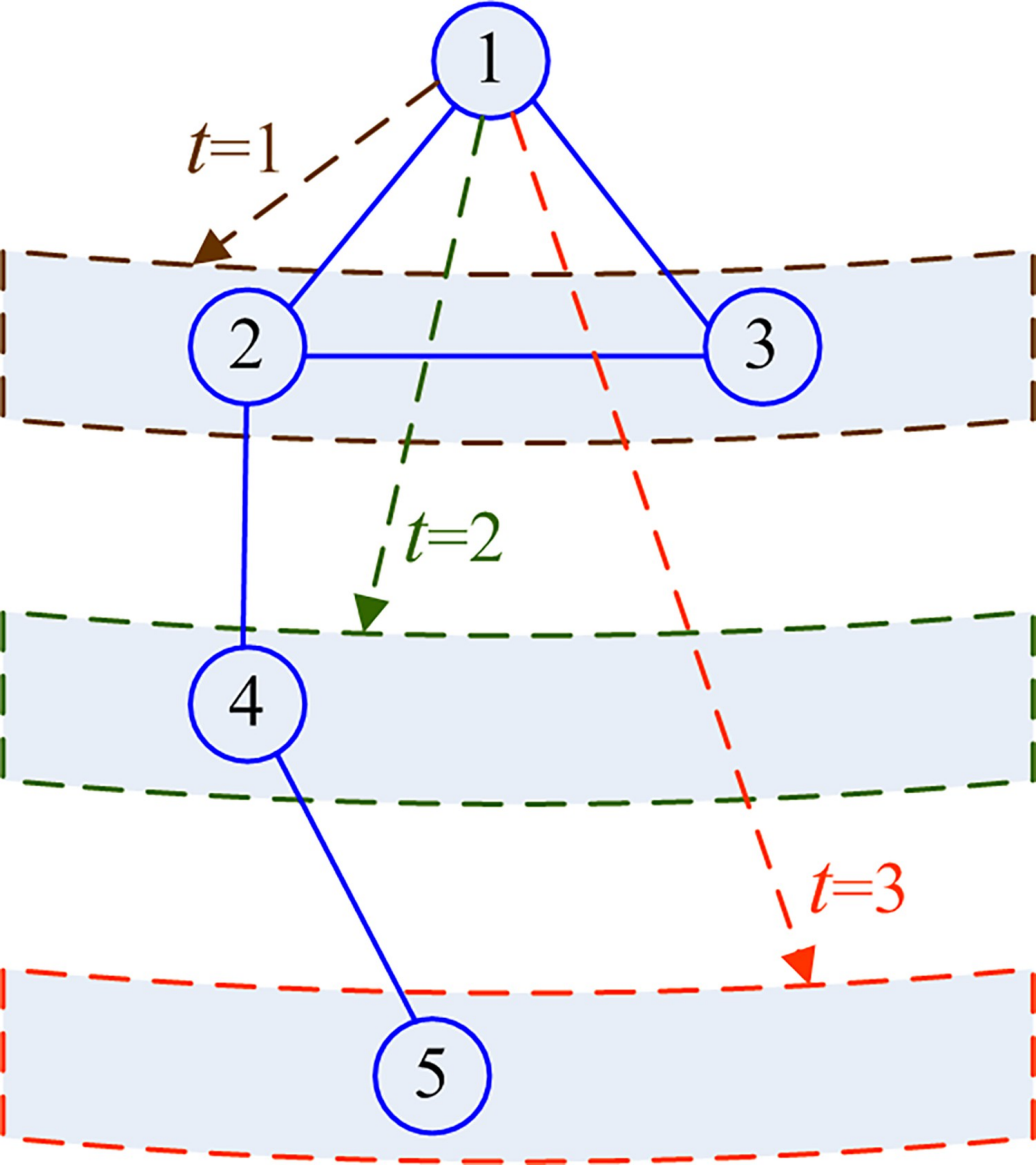
An example of *t-hop* neighbors. The 1*-hop* neighbors of node 1 are the nodes 2 and 3, $N_{1}^{2}=\{2,3,4\}$ and $N_{1}^{3}=\{2,3,4,5\}$.


**Fig 2:**
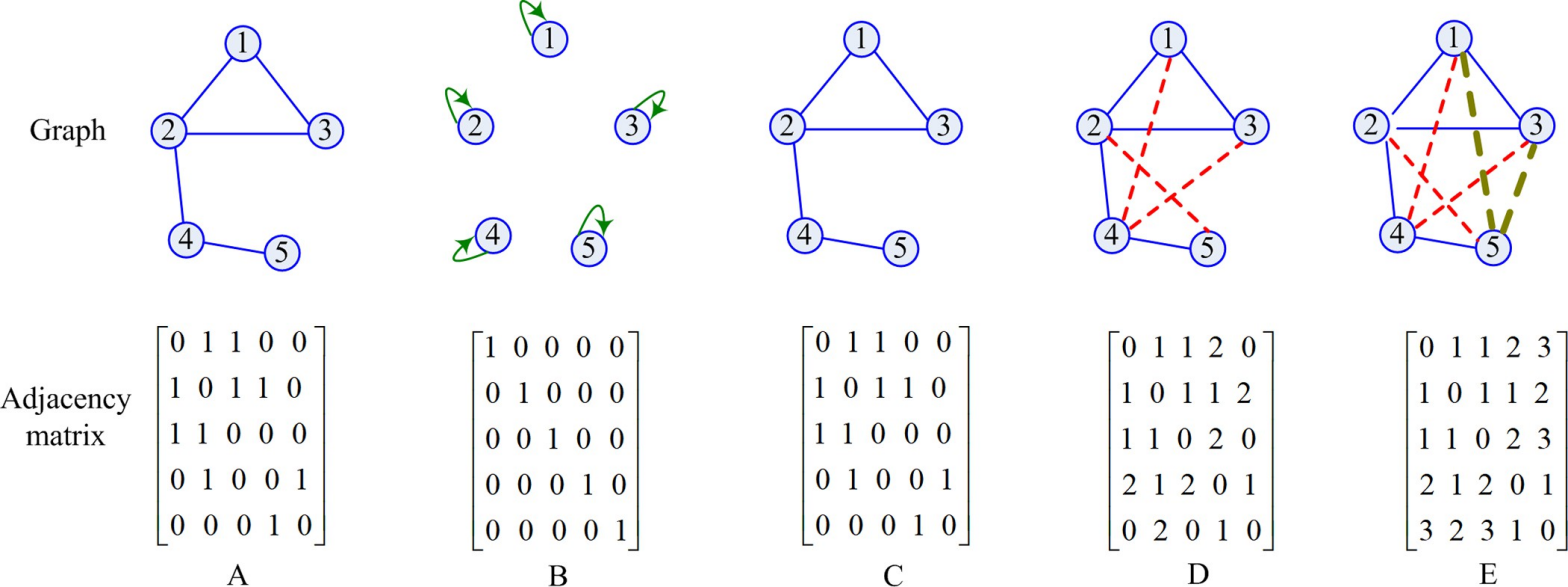
The *t-hop* neighbors and adjacency matrices.

Fig 1 illustrates the *t-hop* neighbors. For example, the 1*-hop* neighbors of node 1 are nodes 2 and 3. Then, we define the adjacency matrix of *t-hop* neighbors on graph (**M**^*t*−*hop*^). In order to enhance the role of path in node feature, the element of **M**^*t*−*hop*^ is set the shortest path between two nodes (see Eq (5), Fig 2). Fig 2A is a graph and its adjacency matrices. Fig 2B illustrates the 0*-hop* neighbors for each node and its adjacency matrix **M**^0−*hop*^. In fact, the adjacency matrix for 0*-hop* neighbors and adjacency matrix (**M**^1−*hop*^) for 1*-hop* neighbors (Fig 2C) are the identity matrix and adjacency matrix of the graph, respectively. Fig 2D and 2E represent the adjacency matrix of 2*-hop* neighbors and adjacency matrix of 3*-hop* neighbors, respectively.


$$N_{i}^{t}=\left\{ \begin{array}{l} {\{v_{j}|v}_{j}\in V,\;i\neq j\;and\;d_{sp}(i,j)\leq t\}\;t>0\\ \{v_{i}\}\;t=0 \end{array}\right.$$ (4)

$$M_{ij}^{t-hop}=\left\{ \begin{array}{l} d_{sp}(i,j)\;i\neq j\\ 0\;i=j \end{array}\right.$$ (5)

To generate a new feature matrix **Y** from **X** by using one layer GCN, we can rewrite the Eq (6) by setting $\hat{A}={\tilde{D}}^{-1/2}\tilde{A}{\tilde{D}}^{-1/2}={\tilde{D}}^{-1/2}(A+I){\tilde{D}}^{-1/2}$, and the Eq (6) can be represented by Eq (7).

$$Y={\tilde{D}}^{-1/2}\tilde{A}{\tilde{D}}^{-1/2}X$$ (6)

$$Y=\hat{A}X$$ (7)

where $\hat{A}$ is a convolutional matrix. Clearly, the graph convolution is the key to the huge performance gain because GCN mixes the features of a vertex and its nearby neighbors [26]. In fact, the role of matrices $\tilde{D}$ and **I** are to normalize the adjacency matrix **A**. Therefore, we can simplify the Eq (7) by replacing $\hat{A}$ with **A** (see Eq (8)).


Y=AX (8)

From Eq (8), we can obtain the value of **Y**_*ij*_ (see Eq (9)) that aggregate the sum of the neighbor’s features.

$$Y_{ij}=\sum_{h}^{N_{i}}A_{ih}\times X_{hj}$$ (9)

where *N*_*i*_ is the neighbors of node *v*_*i*_. If the nodes in the graph do not have features, the feature matrix in GCN is set as identity matrix (Eq (10)).


Y=AI (10)

The Eq (10) can be rewrite by an adjacency matrix of 0*-hop* **M**^0−*hop*^ (see Eq (11)), and the element can be rewrite by Eq (12).


$${Y=AM}^{0-hop}$$ (11)

$$Y_{ij}=\sum_{l}^{N_{ij}^{0}}A_{il}\times M_{lj}^{0-hop}$$ (12)

In Eq (12), $N_{ij}^{0}$ represent the intersection of $N_{i}^{0}$ and $N_{j}^{0},N_{i}^{0}$ and $N_{j}^{0}$ are 0*-hop* neighbors of nodes *v*_*i*_ and *v*_*j*_, respectively.

From Eq (11) and Eq (12), we can see that GCNs only capture self-feature when nodes do not have features. Thus, GCN cannot perform well on the graphs without nodes features. In this work, we use adjacency matrix **M**^*t*−*hop*^ as feature matrix for GCNs because the high hop of **M**^*t*−*hop*^ can capture more information on neighbors. Eq (13) shows the element **Y**_*ij*_ in our method. The feed forward propagation in our method is described as Eq (14).


$$Y_{ij}=\sum_{l}^{N_{ij}^{t}}A_{il}\times M_{lj}^{t-hop}$$ (13)

$$H^{\left(l+1\right)}=\sigma ({\tilde{D}}^{-1/2}\tilde{A}{\tilde{D}}^{-1/2}{\left(M^{t-hop}\right)}^{\left(l\right)}W^{\left(l\right)})$$ (14)

## 3. Results

In order to verify the effectiveness of our method *t-hop*GCN, 12 methods including GCN [5], FastGCN [20], GAT [34], SGC [35], ClusterGCN [19], DAGNN [25], APPNP [36], SSGC [37], GraphMLP [38], RobustGCN [39], LATGCN [40] and MedianGCN [41] are tested on six widely used datasets (see S1 Datasets). These six datasets are Cora, Citeseer and Pubmed [42], Karate [43], Dolphins [44] and Polbook (http://www-personal.umich.edu/~mejn/netdata/, Books about US politics). The former three datasets are citation networks in which each node has label and features. The later three datasets are graphs with strong community structure and the nodes in these graphs have no labels and features. In the study, we treat the nodes in the same community with the same class. For Citeseer graph, we only select the nodes with labels and features, as a results, the Citeseer graph includes 3312 nodes. Table 1 illustrates some characteristics of six graphs. Note that, if the graph is disconnected, the diameter is the maximum of diameters of all connected components, and the average path length is the mean of the average path lengths of all connected components.

**Table 1:** Some characteristics of six graphs.

Graphs	Nodes	Edges	Classes	Diameter	Connectivity
Cora	2708	5429	7	19	No
Citeseer	3312	4732	6	28	No
Pubmed	19717	44338	3	18	Yes
Karate	34	78	2	5	Yes
Dolphins	62	159	2	8	Yes
Polbook	105	441	3	7	Yes

First, the feature matrices (see S2 Datasets) are set as identity matrices for 12 baseline methods and the adjacency matrix of *t-hop* neighbors for *t-hop*GCN. The parameters in the 12 methods are default in GraphGallery [45], which is an easy-to-use platform for fast benchmarking and easy development of graph neural networks. Then, for Cora, Citeseer and Pubmed graphs, the order of the nodes in the feature matrix is the same as the order of the nodes in the original data, and we evaluate *t-hop*GCN and 12 methods with 5% of the training size and 10% of the test size, respectively. For other three small graphs, the order of the nodes in the feature matrices are rearranged by their classes, where we rank the nodes alternately using the labels of classes. Since the sizes of the three graphs are small, we evaluate *t-hop*GCN and 12 methods with 20% of the training size and 20% of the test size, respectively. The accuracy and (weighted) F-score [46] of *t-hop*GCN and 12 methods are shown in Tables 2 and 3.

**Table 2:** The accuracy of different methods.

Methods	Cora	Citeseer	Pubmed	Karate	Dolphins	Polbook
*t-hop*GCN	**0.3630**	**0.4350**	**0.7713**	0.5714	**0.7692**	0.4545
GCN	0.1593	0.1722	0.5289	0.4286	0.3846	**0.5455**
FastGCN	0.2333	0.1390	0.5005	0.5714	0.6154	0.4545
GAT	0.3370	0.1208	0.4894	0.5714	0.3846	0.5000
SGC	0.3000	0.1178	0.5066	0.5714	0.5385	0.4545
ClusterGCN	0.3037	0.1752	0.5132	**0.8571**	0.5385	0.5000
DAGNN	0.2444	0.1601	0.5051	0.5714	0.3846	0.5000
APPNP	0.3148	0.1329	0.5030	0.5714	**0.7692**	**0.5455**
SSGC	0.2926	0.1329	0.5259	0.5714	0.3846	0.4545
GraphMLP	0.1630	0.2356	0.4178	**0.8571**	0.6154	0.4545
RobustGCN	0.2815	0.1118	0.4772	**0.8571**	0.3077	0.5000
LATGCN	0.1370	0.1420	0.5254	0.4286	0.3846	0.2727
MedianGCN	0.1556	0.1269	0.4214	0.4286	**0.7692**	0.4545

**Table 3:** The F-score of different methods.

Methods	Cora	Citeseer	Pubmed	Karate	Dolphins	Polbook
*t-hop*GCN	**0.2875**	**0.3994**	**0.7642**	0.5143	0.6689	0.3961
GCN	0.1723	0.1586	0.5042	0.2571	0.4308	0.5076
FastGCN	0.1671	0.1182	0.4014	0.5143	0.5861	0.4545
GAT	0.1718	0.0900	0.4075	0.5143	0.4308	0.3772
SGC	0.1850	0.0904	0.4652	0.5143	0.5549	0.2841
ClusterGCN	0.2005	0.1595	0.4880	**0.8571**	0.5705	0.4842
DAGNN	0.2287	0.1219	0.4926	0.5143	0.4308	0.3636
APPNP	0.1834	0.1155	0.4395	0.5143	0.6689	0.5455
SSGC	0.2354	0.1142	0.4804	0.5143	0.4066	0.4545
GraphMLP	0.1741	0.2393	0.4436	**0.8571**	0.6584	0.4569
RobustGCN	0.1737	0.0788	0.4136	0.7143	0.2582	**0.5573**
LATGCN	0.1359	0.1234	0.5044	0.2571	0.4359	0.3000
MedianGCN	0.1639	0.0912	0.3865	0.2571	**0.7516**	0.2841

For the Cora, Citeseer and Pubmed without community structure, our method *t-hop*GCN outperforms other methods significantly on accuracy (see Table 2) and F-score (see Table 3). Using accuracy, *t-hop*GCN improves over GCN by 20.37% on Cora, and improves over the worst LATGCN by 22.6% and the best GAT by 2.6%, respectively. On Citeseer, *t-hop*GCN improves over RobustGCN with the worst performance by 32.32% and GraphMLP with the best performance by 19.94%, respectively. For Pubmed, the two relative increases are 35.35% and 24.24%, respectively. Likewise, *t-hop*GCN shows promising results of F-score compared with other methods (see Table 3). For instance, *t-hop*GCN gives 15.16%, 32.06% and 37.77% relative improvements over the worst methods (LATGCN, RobustGCN and MedianGCN) on Cora, Citeseer and Pubmed respectively. The three relative improvements over the best methods (SSGC, GraphMLP and LATGCN) are 5.21%, 16.01% and 25.98%, respectively.

On Dolphins data, *t-hop*GCN achieves the highest accuracy of 76.92%. Using the F-score, the performance *t-hop*GCN is 66.89% slightly lower than MedianGCN with the best performance. For accuracy and F-score, *t-hop*GCN does not perform better than other methods on Karate and Polbook. The potential reason is that the three graphs have strong community structure [47], but *t-hop*GCN cannot capture it well.

## 4. *t-hop* features improve different GNNs

**Fig 3:**
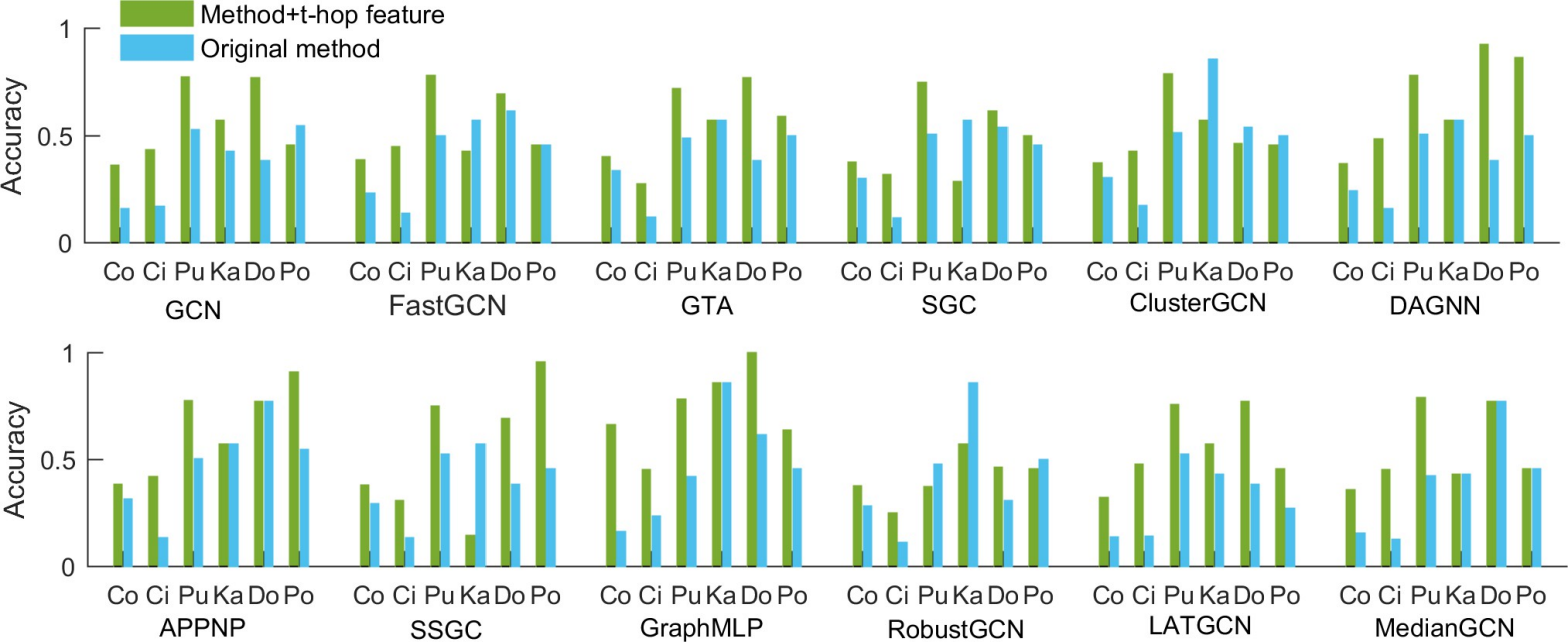
Accuracy comparison between original methods and modified methods with *t-hop* features, Co, Ci, Pu, Ka, Do and Po represent Cora, Citeseer and Pubmed, Karate, Dolphins and Polbook.

**Fig 4:**
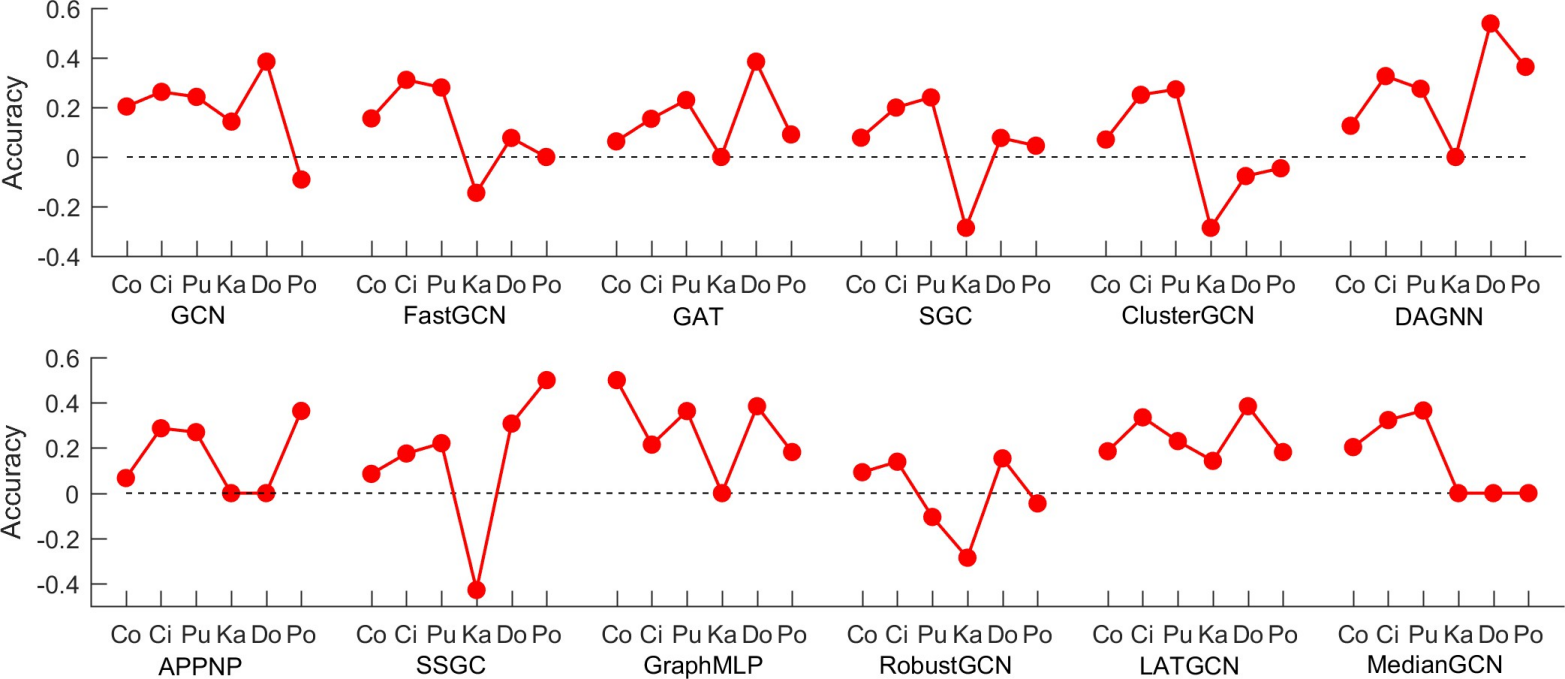
The accuracy improvement or decrease by adding *t-hop* features for 12 methods.

In this section, we investigate if adding *t-hop* features can improve the performance of popular GNNs on node classification. Here, 12 original methods are compared by adding *t-hop* matrix features. Fig 3 and S1 Fig (see Supporting information) show the accuracy and F-score comparison between original methods and the modified methods with *t-hop* feature, and Fig 4 and S2 Fig (see Supporting information) show the accuracy and F-score improvement or decrease by adding *t-hop* features, respectively. From the four figures, we can see that the accuracies and F-scores of 11 methods (except for RobustGCN on Pubmed) are improved remarkably by adding *t-hop* features on Cora, Citeseer and Pubmed. The highest improvements in accuracy and F-score are GraphMLP on Cora data, and the relative increase reaches 50% and 48.84% (see Fig 4 and S2 Fig) respectively. The smallest improvement in accuracy and F-score are GAT on Cora with 6.3% and SGC on Cora with 6.63% respectively.

On karate data with strong community structure, the accuracies of two methods including GCN and LATGCN are improved by 14.28%. The performance of five methods (GAT, DAGNN, APPNP, GraphMLP and MedianGCN) remains the same by adding *t-hop* features, and the rest of five methods yield worse performance after adding *t-hop* features. On the Dolphins data, the accuracies of nine methods are improved, and the best improvement is DAGNN by 53.85%. Unfortunately, the accuracy of ClusterGCN decreases by 7.7% after adding *t-hop* features. For Polbook data, the accuracies of seven methods (GAT, SGC, DAGNN, APPNP, SSGC, GraphMLP and LATGCN) are improved after adding *t-hop* features with a large margin, where the maximal improvement is 50%. However, other three methods perform worse after adding *t-hop* features. Likewise, the performance on F-score has not been improved effectively by adding *t-hop* features on Karate (see S2 Fig), and the F-scores of only three methods are improved. For Dolphins and Polbook, the number of methods whose F-scores are improved significantly by adding *t-hop* features are nine and six, respectively.

**Fig 5:**
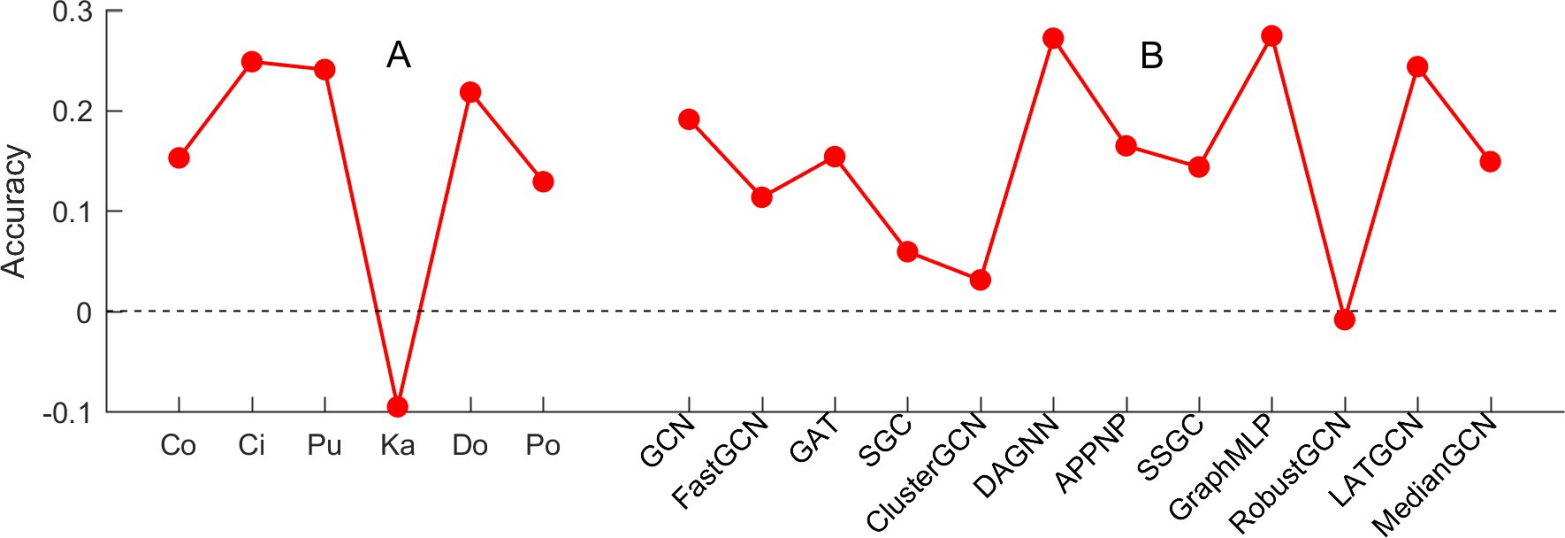
The improvement of average accuracy on six graphs and 12 methods after adding *t-hop* features.

Furthermore, we analyze the average accuracies and F-scores from the points of graph data and methods. Fig 5A and S3A Fig (see Supporting information) show the improvement of the average accuracies and F-scores of different methods on six graphs, and Fig 5B and S3B Fig (see Supporting information) show the improvement of the average accuracies and F-scores on different graphs on 12 methods respectively. As shown in the Fig 5A and S3A Fig, we can see that the average accuracy and F-score on Cora, Cireseer, Pubmed, Dolphins and Polbook are improved significantly by adding *t-hop* features, while the average accuracy and F-score on Karate decreases sharply. The potential reason is that Karate data has strong community structure which contains *t-hop* information. As shown in Fig 5B, we can see that the average accuracies of 11 methods are improved significantly. The best improvement is GraphMLP with a relative increase of 27.4%. Only the method RobustGCN with *t-hop* features achieve worse average accuracy by 0.85%. When we use F-score to measure these results, the average F-scores of all 12 methods are improved and the highest improvement achieves 25.49% (see S3B Fig). These results suggest that our study will open a new idea to research node classification in graphs without features.

### 5. Selection of the parameter *t* in *t-hop*GCN

**Fig 6:**
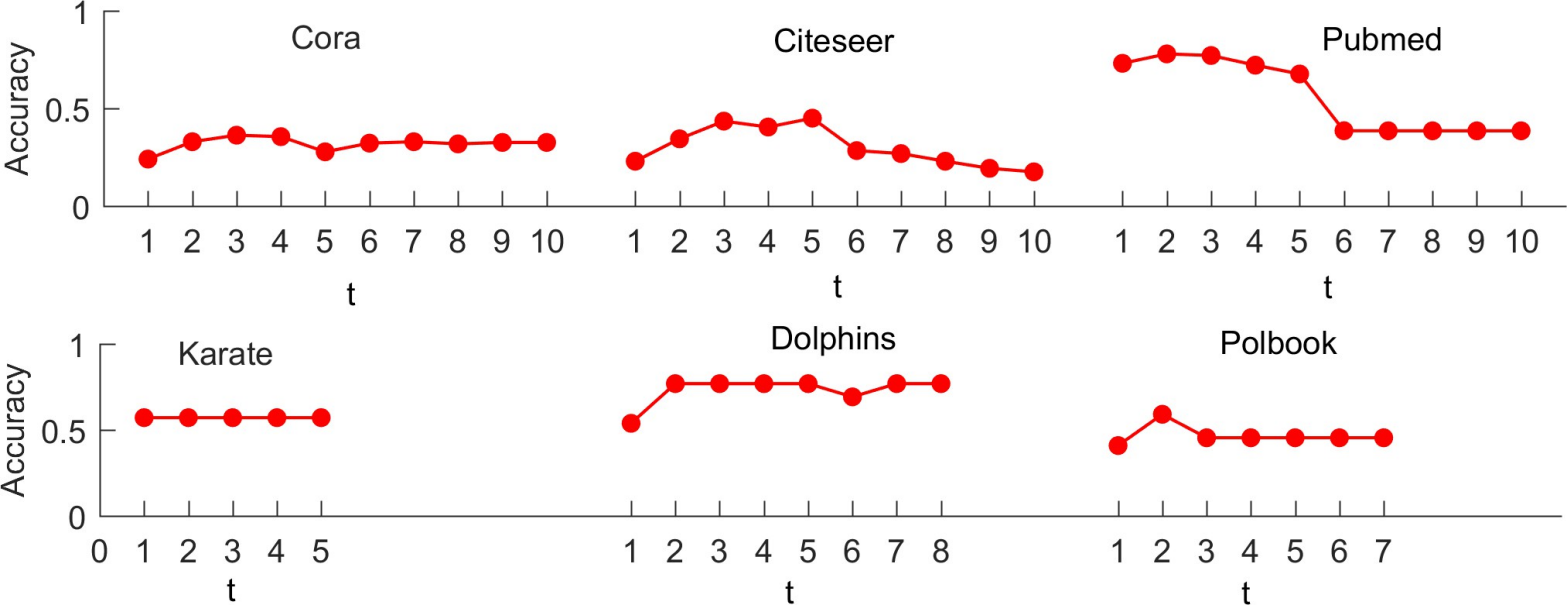
The relationship between the parameter *t* and the accuracy of *t-hop*GCN on six graphs.

The parameter *t* plays a vital role in *t-hop*GCN. Here, we investigate the relationship between the parameter *t* and the accuracy and F-score of *t-hop*GCN. If the graph diameter greater than or equal to 10, the parameter *t* is set from 1 to 10 (see Eq (15)). Otherwise, the parameter *t* is set from 1 to the diameter. Fig 6 and S4 Fig (see Supporting information) show the changes of accuracy and F-score of *t-hop*GCN on six graphs by increasing the parameter *t*. Overall, the accuracy and F-score of *t-hop*GCN decrease as the parameter *t* grows. More specifically, *t-hop*GCN achieves the best performance on accuracy and F-score when *t* = 3 on Cora and Dolphins. Moreover, *t-hop*GCN with *t* = 3 gets the highest value of F-score on Citeseer. *t-hop*GCN achieves the best performance (accuracy and F-score) when *t* = 2 on Pubmed and Polbook. Although the highest value of the accuracy with 77.94% and F-score with 77.62% are appears when the parameter *t* is set as 2 for Pubmed, the *t-hop*GCN with *t* = 3 achieves a close accuracy of 77.13% and F-score of 76.42% respectively. Similarly, although the parameter *t* is set 3, Citeseer with 43.5% accuracy does not achieve the highest value, the difference between the accuracy with *t* = 3 and the best accuracy with *t* = 5 is only 1.52%. For Karate, with the increase in the parameter *t*, the values of the accuracy and F-score remain the same, probably due to strong community structure. In summary, it is reasonable to set the parameter *t* to 3 for *t-hop*GCN, and a smaller parameter *t* can also reduce the computation complexity.


$$t=\left\{ \begin{array}{c} {}[1,2,\cdots ,10]\;diameter\geq 10\\ {}[1,2,\cdots ,diameter]\;diameter<10 \end{array}\right.$$ (15)

### 6. Conclusion

In order to improve the performance of node classification using GCN without node features, we propose a new method named *t-hop*GCN with *t-hop* adjacency matrix as node features. Experimental results show that *t-hop*GCN can significantly improve the performance of node classification on six graphs without node features. For example, on Cora, Citeseer and Pubmed, *t-hop*GCN gains the best accuracy and F-score comparing with other 12 methods, and the best improvements are 35.35% and 37.77%. More importantly, the performance (accuracy and F-score) of 12 GNN methods are improved remarkably by adding *t-hop* features. The highest improvements in accuracy and F-score are GraphMLP on Cora data, and the relative increase reaches 50% and 48.84% respectively. Furthermore, the average accuracies of 11 GNN methods and the average F-scores of 12 GNN methods on six graphs are improved significantly. Thus, the skill for extracting node features from graph structure can be applied to improve the performance of GNNs. It is expected that our research will provide a universal guideline to explore GNNs on the graph without node features for broader potential applications. In future work, we plan to extend the three aspects as following. First, insight into the principle of *t-hop*GCN will be investigated. Second, the relationship between the performance of *t-hop*GCN (or other GNNs methods) and graph structure is still unknown, resulting in poor performance on Karate data by adding *t-hop* features. Third, a pressing problem is to reduce the dimension of the *t-hop* feature matrix that becomes very large and sparse with the increasing size of the graph.

## Supporting information

S1 Datasets Six original graph data. (ZIP)

S2 Datasets Input data for different GNNs. (ZIP)

S1 Fig F-score comparison between original methods and modified methods with *t-hop* features. Co, Ci, Pu, Ka, Do and Po represent Cora, Citeseer and Pubmed, Karate, Dolphins and Polbook.
(TIF)

S2 Fig The F-score improvement or decrease by adding *t-hop* features for 12 methods. (TIF)

S3 Fig The improvement of average F-score on six graphs and 12 methods after adding *t-hop* features. (TIF)

S4 Fig The relationship between the parameter *t* and the F-score of *t-hop*GCN on six graphs. (TIF)
