# Supplementary figures and images for: Path-enhanced graph convolutional networks for node classification without features
Source: PLoS One. 2023 Jun 9;18(6):e0287001. doi: 10.1371/journal.pone.0287001 (PMC10256224; doi:10.1371/journal.pone.0287001)

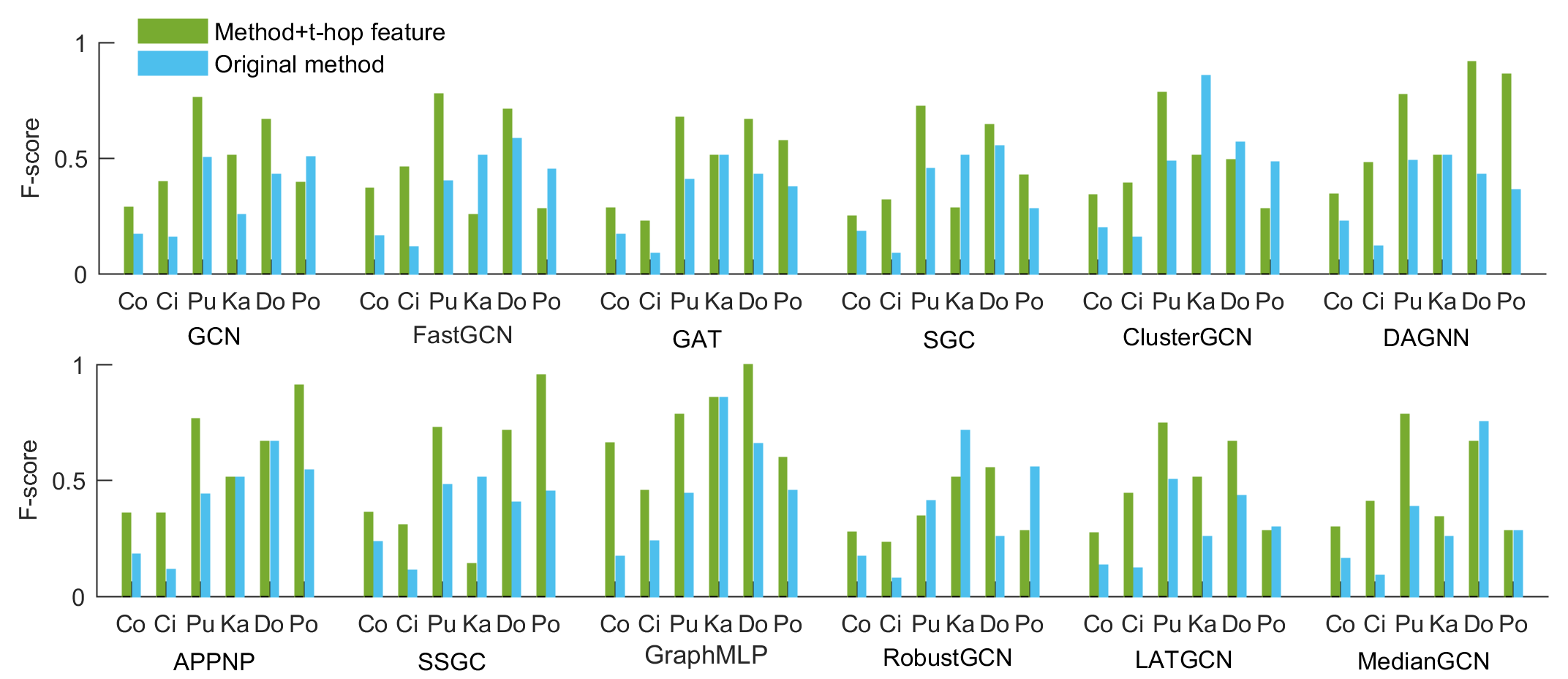

Supplement: S1 Fig — Co, Ci, Pu, Ka, Do and Po represent Cora, Citeseer and Pubmed, Karate, Dolphins and Polbook. (TIF) [file pone.0287001.s003.tif]

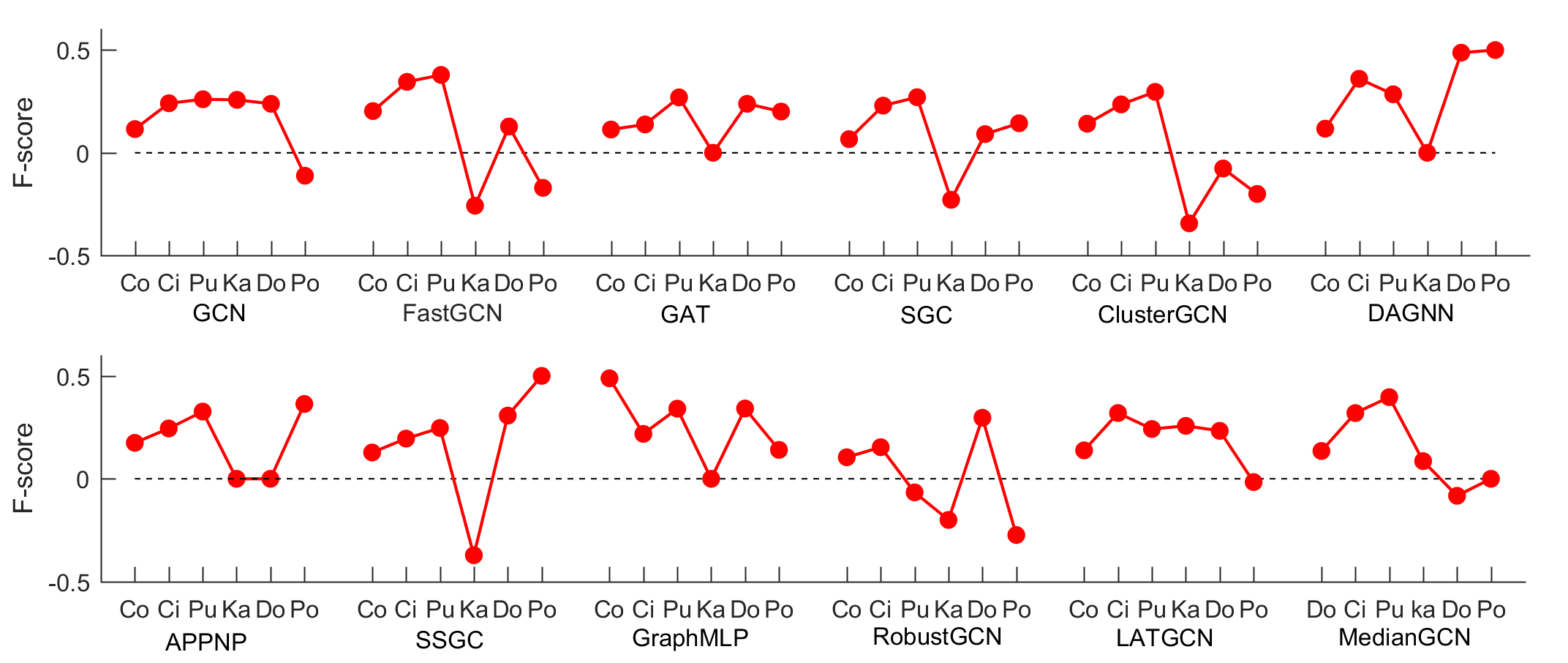

Supplement: S2 Fig — (TIF) [file pone.0287001.s004.tif]

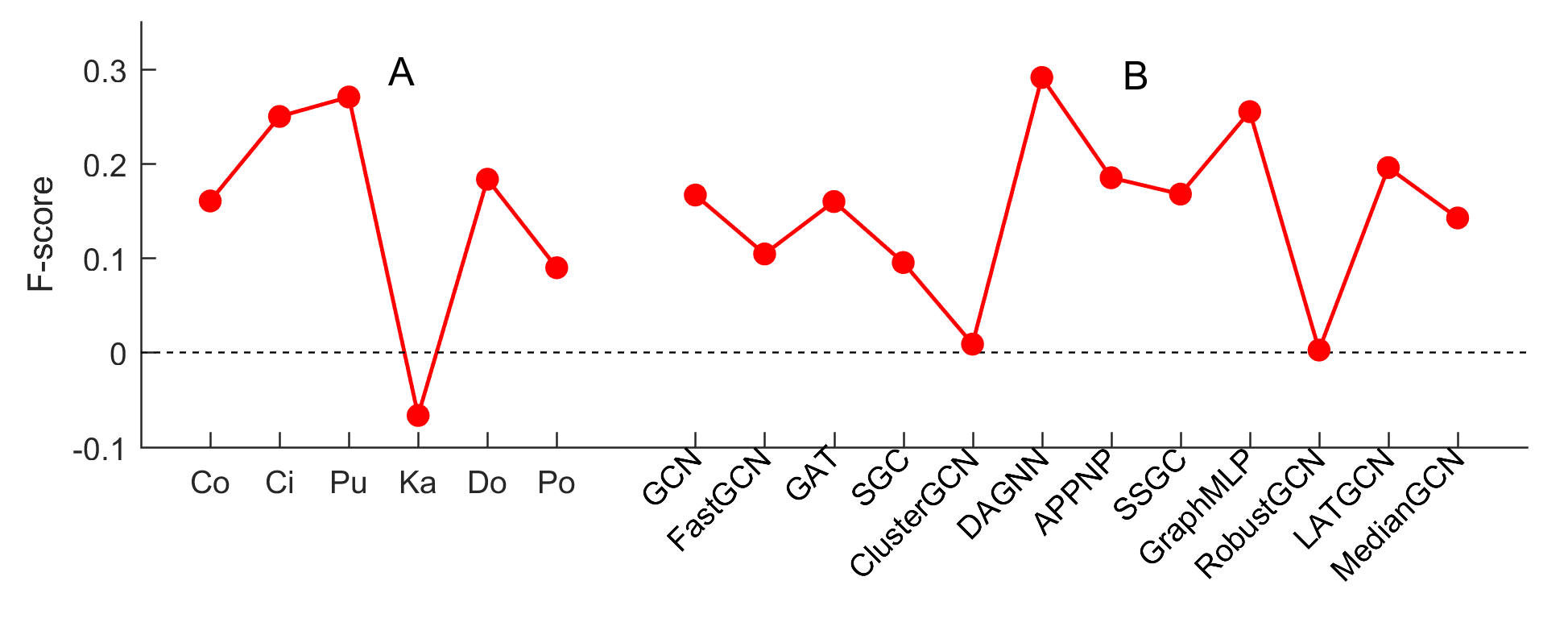

Supplement: S3 Fig — (TIF) [file pone.0287001.s005.tif]

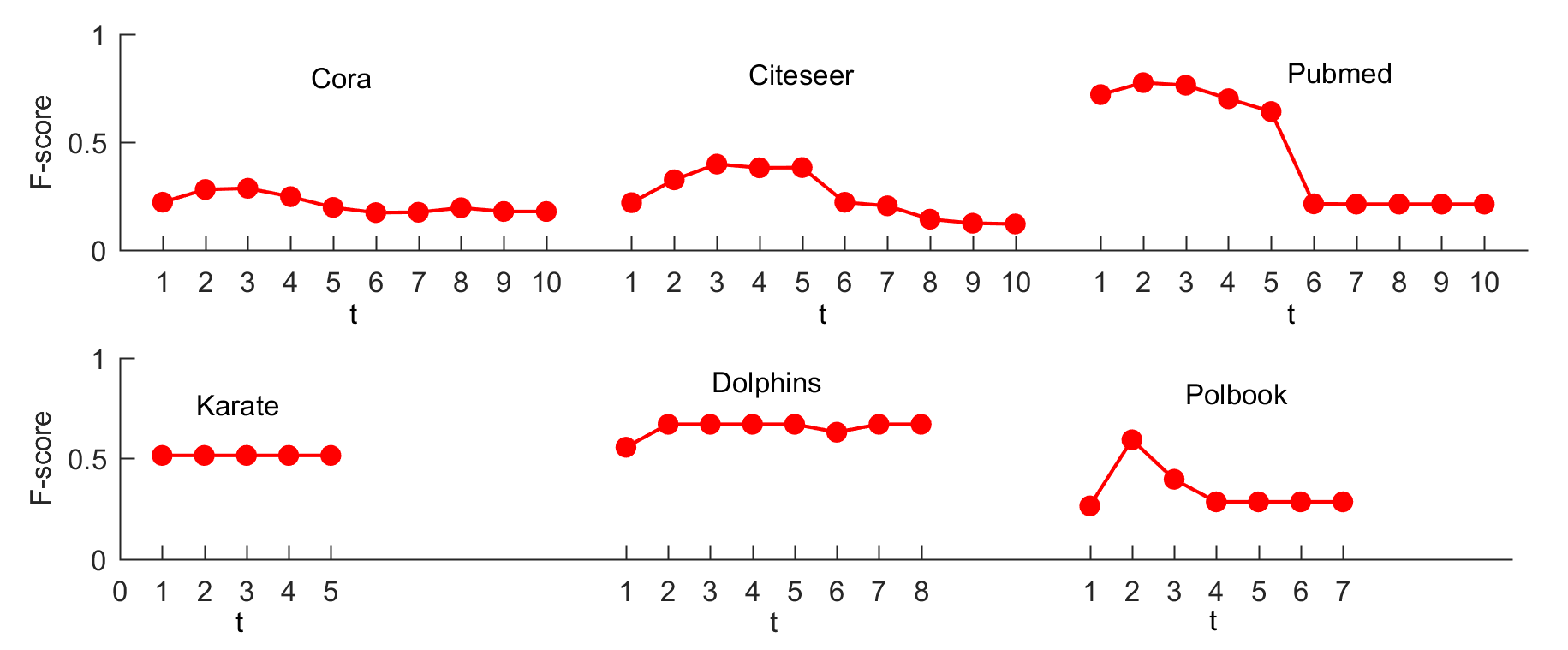

Supplement: S4 Fig — (TIF) [file pone.0287001.s006.tif]
